# Retraction Note: The gastro protective effects of *Cibotium barometz* hair on ethanol-induced gastric ulcer in sprague-dawley rats

**DOI:** 10.1186/s12917-024-04003-0

**Published:** 2024-04-06

**Authors:** Nahla Saeed AL-Wajeeh, Maryam Hajerezaie, Suzita Mohd Noor, Mohammed Farouq Halabi, Nawal Al-Henhena, Ainnul Hamidah Syahadah Azizan, Sareh Kamran, Pouya Hassandarvish, Abdrabuh N. Shwter, Hamed karimian, Hapipah Mohd Ali, Mahmood Ameen Abdulla

**Affiliations:** 1 Department of Biomedical Science, Faculty of Medicine, University of Malaya, Kuala Lumpur, 50603 Malaysia; 2 Department of Microbiology, Faculty of Medicine, University of Malaya, Kuala Lumpur, 50603 Malaysia; 3 Department of Chemistry, Faculty of Science, University of Malaya, Kuala Lumpur, 50603 Malaysia

**Retraction note: BMC Vet Res 13 27 (2016)**

10.1186/s12917-017-0949-z

The Editor has retracted this article after concerns were raised about some of the data reported.

The panel for Fig. 7b appears to overlap with a panel included in a paper with common authors [1] that was under review at the same time as this paper, where the image is described differently.

The authors were unable to provide access to the original data used to construct this figure. The Editor-in-Chief no longer has confidence in the reliability of the article’s findings and conclusions.

Suzita Mohd Noor and Mahmood Ameen Abdulla agree with this retraction. Nahla Saeed AL-Wajeeh, Maryam Hajerezaie, Mohammed Farouq Halabi, Sareh Kamran, Pouya Hassandarvish, Abdrabuh N. Shwter, and Hamed Karimian did not respond to correspondence from the Publisher about this retraction. The Publisher was unable to confirm current contact details for Nawal Al-Henhena, Ainnul Hamidah Syahadah Azizan, and Hapipah Mohd Ali.
